# A Co(II) compound: photocatalytic activity and application value in trigeminal neuralgia with minimally invasive interventional therapy guided by CT

**DOI:** 10.1080/15685551.2022.2115207

**Published:** 2022-08-26

**Authors:** Shu-Wen Wang, Ling-Chao Li

**Affiliations:** a Department of Medical Imaging, Guanyun County People’s Hospital/Guanyun Hospital Affiliated to Kangda College of Nanjing Medical University, Lianyungang, Jiangsu, China; b Department of Pain, The Second Hospital of Dalian Medical University, Dalian, Liaoning, China

**Keywords:** Co(II) compound, photocatalysis, trigeminal neuralgia

## Abstract

A new thermostable Co(II)-based compound, namely [Co_3_(L)_2_(HTEA)_2_]_n_ (**1**, HL = isonicotinic acid, H_3_TEA = triethanolamine), has been successfully synthesized by the isomicotinic acid ligand and HTEA anion. The photocatalytic property of **1** was also investigated, indicating that it shows excellent photocatalytic activity for the degradation of Rhodamine B (MB) solution under the UV light irradiation. For the treatment of trigeminal neuralgia, the content of the inflammatory cytokines released into the trigeminal ganglion tissue fluid was measured with enzyme-linked immunosorbent assay (ELISA) assay. Then, the real-time Reverse Transcription-Polymerase Chain Reaction (RT-PCR) was conducted and the activation of the nuclear factor kappa-B (NF-κB) inflammatory signaling pathway was measured.

## Introduction

Trigeminal Neuralgia (TN) is the most common facial nerve pain, which seriously affects the quality of life of patients. The annual incidence rate of TN is about 12.6/100 000, the incidence is mostly in people over 50 years old, and there are more women than men, with the incidence ratio of women to men is 1.5:1 to 2:1 [1]. At present, TN is widely studied by scholars in various fields, including pathology, neuromorphology, stomatology, ophthalmology, psychiatry, neurology, neurosurgery, etc [2]. But so far, there are many different opinions on the pathogenesis of TN, and most of them are controversial.

With the acceleration of industrialization, organic dyes, widely used in textile industry, paints, paper coloring, leather, etc., have been largely discharged into rivers without any treatment, which will result in serious damages to human life and ecological environment owing to their mutagenic and potentially carcinogenic [3,4]. These dyes feature a common aromatic feature that makes it hard to degrade into harmless small molecules in a short time [5]. Therefore, it is urgent for us to develop an efficient and low-cost strategy to remove organic dyes from industrial wastewater. Recently, photocatalytic technology that used the clear solar energy has been regarded as an ideal sustainable approach for the water purification, more importantly, which produces no secondary pollution and shows high degradation efficiency [6,7]. In recent years, metal-organic frameworks (MOFs) emerged as a new class of hybrid functional materials show excellent photocatalytic activities for the degradation of dye molecules under UV light irradiation [8–11]. For example, Lu *et al.*, employed flexible dicarboxylate ligand and different N-donor ligands to synthesize a series of Co(II)/Cu(II)-based MOFs that show high photocatalytic efficiency for the degradation of methyl violet (MV) under UV light irradiation [12]. Wang et al employed a bifunctional isonicotinic acid ligand to construct a heterometallic Cu(I)-Ca(II) coordination polymer with excellent photocatalytic activity for the photodegradation of MB solution [13].

**Scheme 1.**
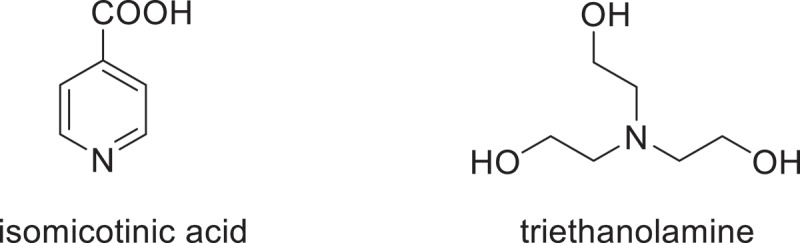
The chemical drawings of the ligands used in this work.

In order to construct new MOFs with photocatalytic activities, in this work, we selected a bifunctional isonicotinic acid ligand, which has been widely used to construct lots of MOFs with multiple functional properties [14,15], to assemble with Co(II) ions with the help of triethanolamine dianions under hydrothermal conditions. Successfully, we obtained a new thermostable Co(II)-based compound, namely [Co_3_(L)_2_(HTEA)_2_]_n_ (**1**, HL = isonicotinic acid, H_3_TEA = triethanolamine, Scheme 1). X-ray crystallographic analysis revealed that compound **1** exhibits a 2D extended layered structure with a linear trinuclear [Co_3_(COO)_2_(HTEA)_2_] cluster subunits. Further layer-to-layer interdigitation induced by weak Van der Waal forces resulted in a 3D supramolecular framework. Furthermore, the photocatalytic activity of **1** was also evaluated by the degradation of MB solution under UV irradiation. Serial experiments were carried out in this research to assess the treatment activity of the compound on the trigeaminal neuralgia treatment.

## Experimental

### Materials and instrumentation

The used reagents in this work are analytical grade and can be available commercially from Jinan Henghua Company. C, H, and N elemental analyses for **1** were performed using an elemental Vario EL III analyzer. Powder X-ray diffraction (PXRD) characterization for **1** was conducted on a PANalytical X’Pert Pro powder diffractometer with Cu/Kα radiation (*λ* = 1.54056 Å) with a step size of 0.05°. Thermogravimetric analysis was performed on a NETSCHZ STA-449C thermoanalyzer with a heating rate of 10°C/min under nitrogen atmosphere from 30°C to 800°C. The photocatalytic experiments were carried out in a Per see TU-1950 UV-vis spectrophotometer.

### Synthesis of compound [Co_3_(L)_2_(HTEA)_2_]}_n_ (1)

A mixture of Co(NO_3_)_2_ · 6H_2_O (0.200 mmol), HL (0.1 mmol), H_3_TEA (0.1 mmol), and H_2_O (12.0 mL) was sealed into a 23 mL Parr Teflon-lined stainless steel vessel, which was heated to 180°C and held at that temperature for 36 h. After cooling to the room temperature at a rate of 2°C/min, purple block crystals of **1** were isolated in 35% yielding based on HL. Elemental analysis is calculated. for C_24_H_34_Co_3_N_4_O_10_ (715.34): C, 40.26; H, 4.75; N, 7.83%. Found: C, 40.24; H, 4.72; N, 7.86%.

### X-ray crystallography

The single crystal data were collected on a Mercury CCD diffractometer equipped with a graphite monochromated Mo–K*α* radiation (*λ* = 0.71073 Å) at room temperature. The structure was solved by the direct method using the ShelXS structure solution program and refined with the ShelXL refinement package using least-squares minimization [16]. Crystal Data for **1**, C_24_H_34_Co_3_N_4_O_10_ (*M* = 715.34 g/mol): monoclinic, space group C2/c (no. 15), *a* = 16.092(4) Å, *b* = 13.371(4) Å, *c* = 13.416(6) Å, *β* = 98.168(6)°, *V* = 2857.4(17) Å^3^, *Z* = 4, *T* = 293.15 K, μ(MoKα) = 1.782 mm^−1^, *Dcalc* = 1.653 g/cm^3^, 10,664 reflections measured (5.114° ≤ 2Θ ≤ 54.98°), 3168 unique (*R*_int_ = 0.0189, R_sigma_ = 0.0171) which were used in all calculations. The final *R*_1_ was 0.0660 (I > 2σ(I)) and *wR*_2_ was 0.1854 (all data). CCDC number: 2,101,330.

### ELISA assay

The ELISA assay was conducted in this present research to evaluate the inhibitory activity of the compound on the content of the inflammatory cytokines released into the trigeminal ganglion tissue fluid. This preformation was conducted totally under the guidance of the instructions with only a little change. In brief, 50 SD rats used in this present research were obtained from the Changchun Jingcheng Model Animal Technology Co., Ltd. All the conduction in this research was approved by the China Animal Ethics Committee. Before the compound treatment, the animals were cultured in a standard condition of 20–25°C, and then the animals were divided into the control group, model group, and compound treatment groups. The trigeminal ganglion animal model was constructed, followed by the compound treatment at the concentration of 1, 2, and 5 mg/kg. Finally, the trigeminal ganglion tissue fluid was collected and the content of inflammatory cytokines was measured.

### Real time RT-PCR

To assess the activation of the NF-κB inflammatory signaling pathway, the real-time RT-PCR was also conducted in this research. This experiment was carried out strictly in accordance with the protocols with only a small change. In short, 50 SD rats used in this present research were obtained from the Changchun Jingcheng Model Animal Technology Co., Ltd. All the conduction in this research was approved by the China Animal Ethics Committee. Before the compound treatment, the animals were cultured in a standard condition of 20–25°C, and then the animals were divided into the control group, model group, compound treatment groups. The trigeminal ganglion animal model was constructed, followed by the compound treatment at the concentration of 1, 2, and 5 mg/kg. Next, the neurons were collected and the total RNA in the tissue was extracted with TRIZOL reagent. After measuring the concentration of the total RNA, which was then reverse transcribed into cDNA. In the end, the relative expression of the NF-κB inflammatory signaling pathway was measured with real-time RT-PCR, with *gapdh* used as the internal control.

## Results and discussion

### Crystal structure of compound 1

**Figure 1.**
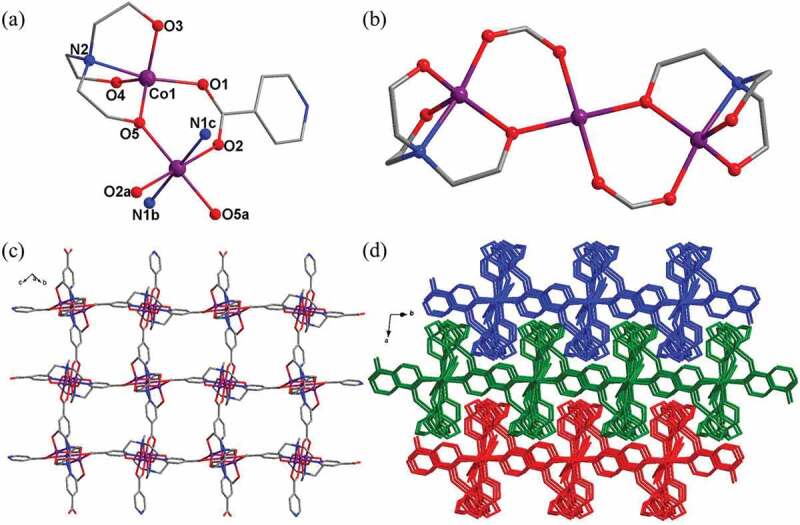
(a) Viewing of the coordination environments of Co(II) ions in **1**. (b) The trinuclear [Co_3_(COO)_2_(HTEA)_2_] cluster. (c) The 2D layered framework of **1**. (d) The interdigitated 3D supramolecular framework for **1.**

Single crystal X-ray structural analysis revealed that the asymmetric unit of **1** contains one and a half Co(II) ions, one L^−^ ligand, as well as a coordinated HTEA anion. As shown in Figure 1(a), Co1 is five-coordinated by one carboxylate oxygen from one L^−^ ligand, three oxygen donors, and one nitrogen donor from one HTEA anion, showing a slightly distorted triangular bipyramidal geometry, while Co2 is six-coordinated by two oxygen donors from two individual HTEA anions, two carboxylate oxygen atoms, and two nitrogen atoms from four different L^−^ ligands, and the coordination geometry of Co2 can be described as a slightly distorted octahedron. Each HTEA anion bonds to two Co(II) ions with two oxygen donors and one nitrogen donor in chelating mode and the other oxygen donor in bridging mode. As a result, two symmetry-related Co1 ions and one Co2 ion are bridged together by two HTEA anions and two bis-monodentate carboxylate groups offered by L^−^ ligands, leading to the formation of a linear trinuclear [Co_3_(COO)_2_(HTEA)] cluster (Figure 1(b)). In this trinuclear cluster, the adjacent Co ^…^ Co distance is 3.62 Å. Each trinuclear cluster connects to four adjacent ones through four L^−^ ligands, and this connection mode further results in the formation of D-layered structure extending along the crystallographic *bc* plane (Figure 1(c)). Viewing along c axis, there exist large voids between the adjacent HTEA anions in the 2D layer, and by the calculation of PLATON program, no hydrogen-bond interactions can be found. Thus, these 2D layers finally interdigitated together in -ABAB- mode under weak Van der Waals interactions, forming an interdigitated 3D supramolecular framework (Figure 1(d)).

### Powder X-ray diffraction pattern (PXRD) and thermogravimetric analysis (TGA)

**Figure 2.**
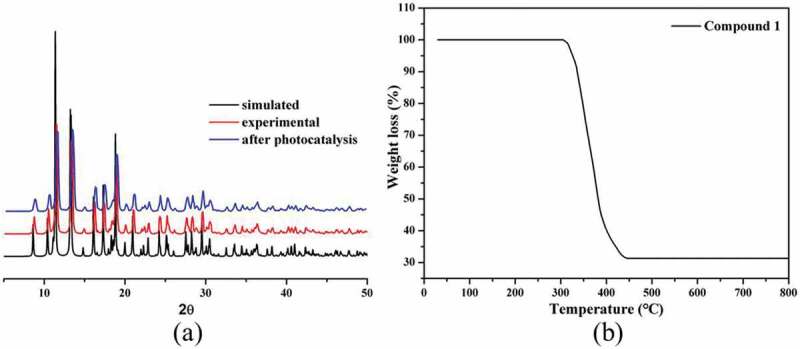
(a) The PXRD patterns for **1**. (b) The TGA curve of **1.**

Powder X-ray diffraction technique was employed to check whether the single structure is representative of the bulk sample. As shown in Figure 2(a), the diffraction peaks from the experimental pattern show good match with those of the pattern simulated from the single crystal data, which demonstrates that the bulk samples of **1** are in single phase.

The result of TGA experiment for **1** is shown in Figure 2(b), and obviously, the framework of **1** shows high thermal stability with no observed weight loss before 305°C. In the temperature range of 305–445°C, compound **1** underwent a rapid and significant weight loss process, which was caused by the pyrolysis of the organic ligands. Finally, the remaining undefined powder samples may be the CoO (obsd: 31.33%, calcd: 31.42%).

### Photocatalytic property of 1

**Figure 3.**
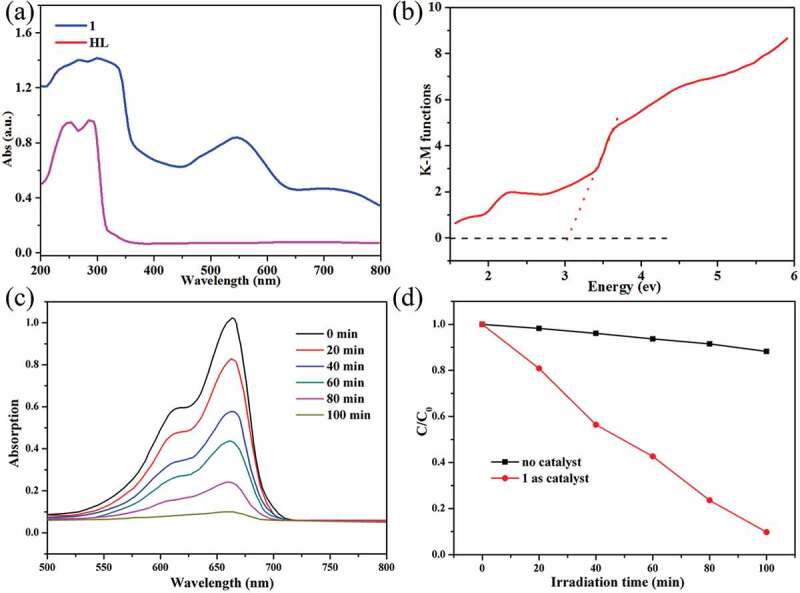
(a) UV-vis absorption spectra of **1** and the free ligands in the solid state; (b) diffuse reflectance spectra of Kubelka–Munk functions *versus* energy (eV) for **1**. (c) The time-dependent UV–vis absorption spectra for the degradations of MB solution in the presence of **1** as photocatalyst. (d) The plots of C/C_0_ versus irradiation time in the presence or absence of **1.**

In order to elucidate the photoresponse region, the solid state UV-vis diffuse reflection (DRS) spectra of **1** and the free organic ligand isonicotinic acid were measured at room temperature (Figure 3(a)). The maximum UV absorption bands for the isonicotinic acid ligand were at *ca*. 254 and 296 nm, which may be attributed to the π → π* transition. In Figure 3(a), two absorption components in both the UV and visible regions for **1** were observed. The UV absorption band (*ca*. 275 nm) can be attributed to the ligand-to-metal charge transfer (LMCT), while the visible absorption band (*ca*. 548 nm) may be due to the d–d transition of the Co^2+^ ions. In previous investigations, the band gap energy (*E*_g_) was one of the key factors evaluating the degradation efficiency of the photocatalysts. Thus, the Kubelka–Munk (K–M) equation, *F* = (1 − *R*)^2^/2 *R* (where *R* is the reflectance of an infinitely thick layer at a given wavelength), was used to calculate the band gap energy (*E*_g_). The *E*_g_ value of **1** was estimated to be 3.08 eV (Figure 3(b)), suggesting that **1** may be responsive to UV light and has potential for photocatalytic reactions. Based on the reports, Co(II)-based MOFs as photocatalysts usually exhibit excellent photocatalytic activities for the degradation of organic dye contaminants, such as methyl blue (MB), methyl orange (MO), methyl violet (MV), Rhodamine B (RhB), etc., under UV light irradiation [17,18]. In view of that, in this work, we also investigated the photocatalytic property of **1** by photocatalytic degradation of MB under UV light irradiation. 50 mg powders of **1** were added to the 100 mL 10 mg/L MB solution, and the solution was further stirred for 1 h under dark to establish adsorption equilibrium between the catalyst and MB solution, and then, this suspension was exposed to UV light irradiation. At a given interval of 20 min, we took out 5 mL reaction mixture periodically, which was further analyzed by the UV-Vis spectrometer. *Via* the time-dependent absorption spectra of MB solution in the presence of **1**, it can be observed that the absorption intensities of the MB characteristic peaks at 664 nm decreased gradually with the increase exposure time of UV light (Figure 3(c)), and the degradation ratio of MB in solution is 90.2% when exposed to UV light for 100 min (Figure 3(d)). A comparative experiment in the absence of **1** was also conducted under the same UV light irradiation, and the degradation ratio of MB in solution is 11.7% after the same irradiation time (Figure 3(b)). Furthermore, the adsorption capacity of **1** was studied under the same conditions without UV irradiation, and it could be found that complex **1** could not adsorb MB dye which might be due to its nonporous structure (Fig S1). Therefore, compound **1** can be served as good photocatalyst for the water purification. After the photocatalytic reaction, the framework of **1** demonstrated by the PXRD characterization is stable (Figure 2(a)). Compared to the previous Co(II) compounds, compound **1** reported here shows better degradation efficiency for MB solution [19].

The activity of the recycled photocatalysts is a very important factor for determining its performance. To evaluate the reusability of these photocatalysts, repeated experiments were also conducted under the same experimental conditions. After the photocatalytic experiment, the photocatalysts were centrifuged to obtain recyclable samples, and the experiment was repeated four times with the recovered sample. The results after five experiments showed that degradation efficiencies of recovered catalysts were not significantly different from the as-synthesized (Fig S2).

### Compound significantly reduce the inflammatory cytokines released into the trigeminal ganglion tissue fluid

**Figure 4.**
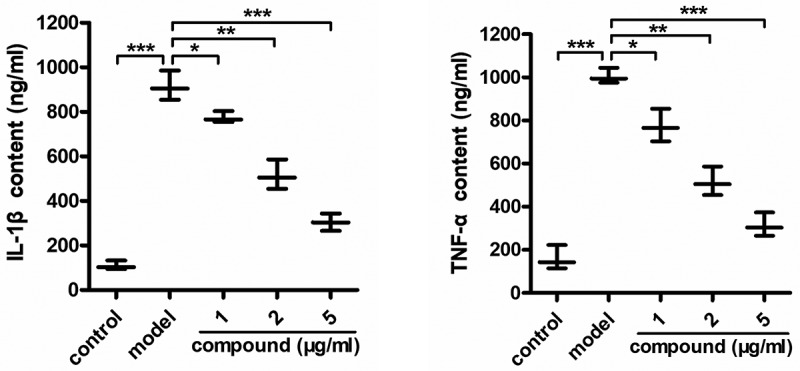
Significantly reduced inflammatory cytokines released into the trigeminal ganglion tissue fluid after compound treatment. The trigeminal ganglion animal model was constructed and the compound was given for treatment at the concentration of 5 mg/kg. The content of the inflammatory cytokines released into the trigeminal ganglion tissue fluid was measured with ELISA assay.

Some scholars believe that trigeminal neuralgia is caused by the overplastic inflammatory response of neuronal cells. Thus, in this present research, the ELISA assay was conducted to determine the content of inflammatory cytokines released into the trigeminal ganglion tissue fluid. As the results showed in Figure 4, we can see that there was a higher level of inflammatory cytokines in the trigeminal ganglion tissue fluid of the model group, compared with the control group. After the treatment of the compound, the inflammatory cytokines content released into the trigeminal ganglion tissue fluid was significantly inhibited. The inhibition of the compound exhibited a dose relationship.

### Compound obviously inhibited the activation of the NF-κB inflammatory signaling pathway

**Figure 5.**
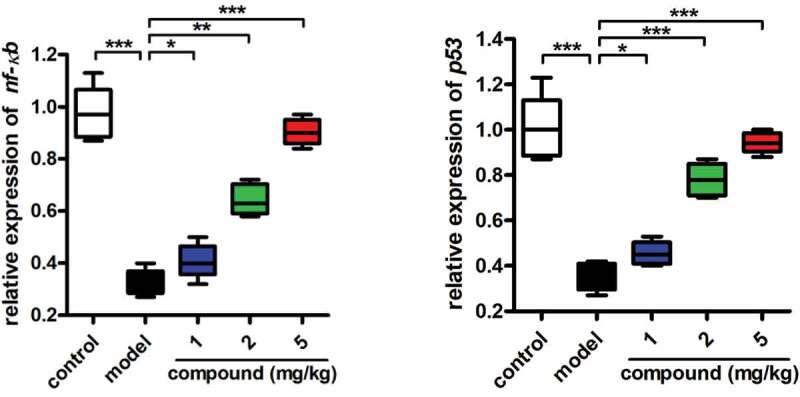
Obviously inhibited activation of the NF-κB inflammatory signaling pathway after compound treatment. The trigeminal ganglion animal model was constructed and the compound was given for treatment at the concentration of 5 mg/kg. Real time RT-PCR was conducted and the activation of the NF-κB inflammatory signaling pathway was measured.

In the previous study, we have proved that the compound has excellent application value in the trigeminal ganglion treatment by inhibiting the content of inflammatory cytokines released into the trigeminal ganglion tissue fluid. As the NF-κB inflammatory signaling pathway regulates the release of inflammatory cytokines, the real-time RT-PCR was further conducted and the activation of NF-κB inflammatory signaling pathway was also determined. The results in Figure 5 suggest that the NF-κB inflammatory signaling pathway activation was much stronger in the model group than in the control group, with P < 0.005. The compound could obviously reduce the NF-κB inflammatory signaling pathway activation dose dependently.

## Conclusion

In summary, we have synthesized a new thermostable Co(II) compound with 2D extended layered structure based on linear trinuclear [Co_3_(COO)_2_(HTEA)_2_] clusters. Under the guidance of weak Van der Waals forces, these 2D layers are interdigitated into a 3D supramolecular framework. The investigation of photocatalytic property indicated that **1** exhibits excellent photocatalytic activity for the UV-light-driven degradation of MB. The results of the ELISA assay indicated that the compound could inhibit the content of the inflammatory cytokines released into the trigeminal ganglion tissue fluid. In addition to this, the activation of the NF-κB inflammatory signaling pathway was obviously suppressed by the compound. Finally, we came to this conclusion: the compound has excellent application value on the rigeaminal neuralgia treatment by inhibiting the inflammatory response.

## Supplementary Material

Supplemental Material

## Data Availability

The adsorption spectra of MB solution without UV irradiation in the presence of 1 (Fig. S1); Cycling runs of **1** in the degradation of the MB solution (Fig. S2), the information could be found in the supporting information file.
