# Supplementary material for: A Co(II) compound: photocatalytic activity and application value in trigeminal neuralgia with minimally invasive interventional therapy guided by CT
Source: Des Monomers Polym. 2022 Aug 26;25(1):254–60. doi: 10.1080/15685551.2022.2115207 (PMC9423858; doi:10.1080/15685551.2022.2115207)
Supplement: Supplemental Material [file TDMP_A_2115207_SM5511.pdf]

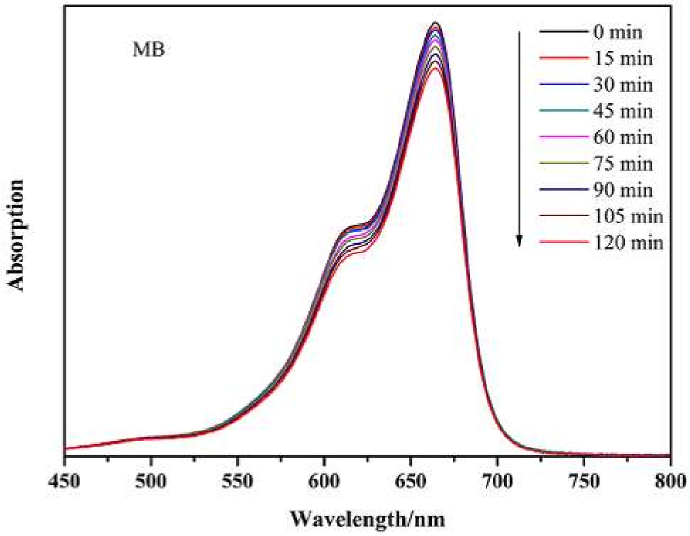

Fig. S1 The adsorption spectra of MB solution without UV irradiation in the presence of **1**.

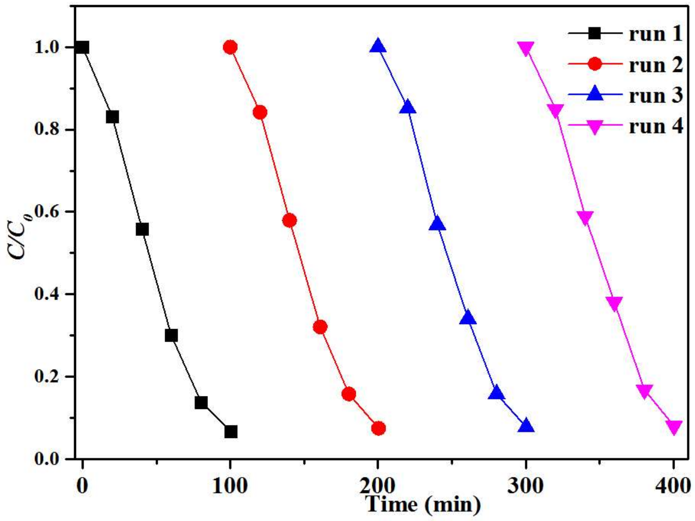

Fig. S2 Cycling runs of **1** in the degradation of the MB solution.
